# Effects of Receptor Specificity and Conformational Stability of Influenza A Virus Hemagglutinin on Infection and Activation of Different Cell Types in Human PBMCs

**DOI:** 10.3389/fimmu.2022.827760

**Published:** 2022-03-14

**Authors:** Jens Dorna, Andreas Kaufmann, Viktoria Bockmann, Hartmann Raifer, Johanna West, Mikhail Matrosovich, Stefan Bauer

**Affiliations:** ^1^ Institute for Immunology, Philipps University Marburg, Marburg, Germany; ^2^ Core Facility FACS, Philipps University Marburg, Marburg, Germany; ^3^ Institute of Virology, Philipps University Marburg, Marburg, Germany

**Keywords:** influenza, hemagglutinin, receptor specificity, primary immune cells, tropism

## Abstract

Humans can be infected by zoonotic avian, pandemic and seasonal influenza A viruses (IAVs), which differ by receptor specificity and conformational stability of their envelope glycoprotein hemagglutinin (HA). It was shown that receptor specificity of the HA determines the tropism of IAVs to human airway epithelial cells, the primary target of IAVs in humans. Less is known about potential effects of the HA properties on viral attachment, infection and activation of human immune cells. To address this question, we studied the infection of total human peripheral blood mononuclear cells (PBMCs) and subpopulations of human PBMCs with well characterized recombinant IAVs differing by the HA and the neuraminidase (NA) but sharing all other viral proteins. Monocytes and all subpopulations of lymphocytes were significantly less susceptible to infection by IAVs with avian-like receptor specificity as compared to human-like IAVs, whereas plasmacytoid dendritic cells (pDCs) and myeloid dendritic cells were equally susceptible to IAVs with avian-like and human-like receptor specificity. This tropism correlated with the surface expression of 2-3-linked sialic acids (avian-type receptors) and 2-6-linked sialic acids (human-type receptors). Despite a reduced infectivity of avian-like IAVs for PBMCs, these viruses were not less efficient than human-like IAVs in terms of cell activation as judged by the induction of cellular mRNA of *IFN-α, CCL5, RIG-I*, and *IL-6*. Elevated levels of IFN-α mRNA were accompanied by elevated IFN-α protein secretion in primary human pDC. We found that high basal expression in monocytes of antiviral interferon-induced transmembrane protein 3 (IFITM3) limited viral infection in these cells. siRNA-mediated knockdown of IFITM3 in monocytes demonstrated that viral sensitivity to inhibition by IFITM3 correlated with the conformational stability of the HA. Our study provides new insights into the role of host- and strain-specific differences of HA in the interaction of IAVs with human immune cells and advances current understanding of the mechanisms of viral cell tropism, pathogenesis and markers of virulence.

## Introduction

Influenza A viruses (IAVs) are a major concern for human health and health care system. From wild aquatic birds, which are the natural reservoir, IAVs can occasionally transmit to different avian and mammalian species such as domestic birds and pigs. Circulation and adaptation in these intermediate hosts can lead to transmission to humans. Although zoonotic IAVs (e.g. H5N1 and H7N9) usually do not transmit among humans, they often cause severe and fatal diseases characterized by an aberrant immune reaction. On very rare occasions, animal viruses can adapt to humans, acquiring the ability of efficient human-to-human transmission, and initiate global pandemics in the immunologically native human population. Pandemic viruses continue to circulate and evolve in the human population causing seasonal influenza outbreaks [for recent reviews on ecology, evolution and pathogenicity of IAVs, see refs (1–5)]. Thus, humans can be infected by avian-origin zoonotic, pandemic and seasonal IAVs, which differ by their level of adaptation to human host and have distinguishable characteristics.

Particularly distinctive are properties of the viral envelope glycoprotein HA of zoonotic avian IAVs and human-adapted pandemic and seasonal IAVs (6–8). The HA is responsible for two critical functions in the viral replication cycle, attachment to sialic acid-containing receptors on host target cells and fusion between viral and endosomal membranes [for reviews, see refs (9–11)]. N-acetylneuraminic acid (Neu5Ac) is the predominant sialic acid (SA) species in birds and humans (12). IAVs can distinguish between Neu5Ac2-6Gal-terminated oligosaccharide receptors (2–6) having a relaxed “open-umbrella” like structure and Neu5Ac2-3Gal-terminated receptors (2, 3) having a closed “cone-like” structure (13). Avian IAVs preferentially bind to Neu5Ac2-3Gal-containing (“avian-type”) receptors abundantly expressed on the surface of avian intestinal epithelium, a major site of IAV replication in birds. Swine- and human-adapted IAVs preferentially bind to Neu5Ac2-6Gal-containing (“human-type”) receptors. These receptors are dominantly expressed in the human upper respiratory tract, facilitating efficient access, replication and human-to-human transmission of human IAVs by airway droplets. By contrast, the Neu5Ac2-3Gal-containing glycans are dominantly expressed in human bronchioles and alveoli. Infection of these tissues with a high viral load and under conditions of inadequate immune response can result in a severe disease but is not compatible with efficient airborne transmission of the virus (14, 15).

Following the attachment and endocytosis of IAVs, the particles are transported *via* the endosomal-lysosomal pathway in which they are exposed to a gradually decreasing pH. This triggers the conformational transition of the HA leading to fusion between the viral and endosomal membrane and subsequent release of viral nucleocapsids into to the cytoplasm. The conformational stability of the HA determines, on the one hand, the pH optimum of fusion (pH range in which the membrane fusion takes place) and, on the other hand, the viral resistance to inactivation by environmental factors (16, 17). Recent studies indicate that the pH optimum of membrane fusion differs among IAVs from different host species and represents, in addition to viral receptor –binding specificity, a determinant of the viral host range and cell tropism (10, 18, 19).

Many studies seek to describe differences in pattern of viral binding, replication and regulation of cytokine production upon infection of avian and human IAVs in the human respiratory tract and upon interactions of IAVs with immune cells. Current models suggest that receptor specificity is the main factor determining tropism of human IAVs to airway epithelial cells in the upper respiratory tract (trachea and bronchi), whereas avian IAVs more efficiently bind and infect non-ciliated epithelial cells, type II pneumocytes and alveolar macrophages in the lower respiratory tract (bronchioles and alveoli) (20, 21). In accord with this concept, avian viruses, such as H5N1 and H7N9, with zoonotic infection potential in humans (5, 22, 23) replicate in deeper parts of the lung. The virus-mediated disruption of epithelial barrier may lead to systemic IAV infection (24–26). In this setting, infection of endothelial cells seems to be dependent on HA stability rather than on receptor specificity (27). Although the effects of different HA on IAV interactions with epithelial cells is well studied, information on interactions with immune cells is limited. In macrophages and DCs, which represent resident cell populations in the lung, recognize IAVs and participate in the initiate immune reaction, the binding of IAVs was shown to be independent from the receptor specificity (28). Although binding of IAV to these cells was independent of the HA receptor specificity, Ramos et al. showed that alteration of the receptor specificity of the H5N1 virus was sufficient for changing the cytokine profile in the infected human monocytes and DCs, indicating different ways of recognition after viral entry to the cells. Moreover the profile of released cytokines seemed to be independent of viral replication (29). In addition, it was shown that efficient replication in these cells was mainly determined by the ability to overcome different intracellular blocking steps associated with conformational stability of HA (30–34). Given the possibility of a systemic infection by IAVs they can occasionally get access to lymphocytes. It remains to be explored whether and how receptor specificity and conformational stability of the HA can affect IAVs attachment, infection and activation of lymphocytes. A limited number of studies reported binding of IAVs to human PBMCs (35, 36). Based on studies in 1980s it was suggested that the infection of lymphocytes requires interaction with monocytes (36–39). A more recent study by Lee and colleagues reported that infection of lymphocytes may depend on viral strain and vary substantially. Within this study, the detailed mechanism for the tropism could not be solved. Also availability of sufficient primary human resident cells as well as utilization of wild type viruses makes the system even more complex (35).

We previously showed that recombinant 2:6 IAVs containing the six internal genes segments of the laboratory strain A/Puerto Rico/8/1934 (PR8) and the respective wild type HAs and NAs of the A/Vietnam/1203/2004 (H5N1), A/Hong Kong/1/1968 (H3N2), and A/Memphis/14/1996 (H1N1) strains, differed by both receptor-binding properties and conformational stability/pH optimum of membrane fusion (27, 40–42). The virus with avian-origin HA (VN) displayed a typical avian-virus-like receptor-binding specificity, binding to type 2,3-linked SA but not to 2,6-linked SA. The virus with the HA of the seasonal IAV (Mem) has a typical human-virus-like specificity and bound exclusively to human-type 2,6-linked SA. The pandemic-origin virus (HK) preferentially bound to human-type receptors, although still preserved some of the binding avidity of its avian precursor for 2,3-linked SA. As typical for the human-adapted viruses, Mem and HK had relatively high conformational stability and underwent acid-induced conformational transition at pH 4.7 and 4.9, respectively. In contrast, poultry-adapted VN was relatively unstable and displayed conformational transition at a much higher pH (5.7) (27, 40, 41).

We hypothesize that receptor specificity and conformational stability of the HA can influence infection/activation of immune cells in early time points of infection. As a result, the susceptibility of cells and/or their activation will differ between immune cell types. We tested these hypotheses using human PBMCs and a set of 2:6 recombinant viruses that differed by either receptor specificity or conformational stability.

## Material and Methods

### Cells

Primary human peripheral blood mononuclear cells (PBMCs) were isolated from healthy voluntary blood donors obtained from the Institute for Clinical Immunology and Transfusion Medicine, Justus-Liebig-University Giessen. Buffy coats were diluted 1:1 with PBSdef (PBS w/o Mg^2+^, Ca^2+^). After separation by density gradient centrifugation with Ficoll Histopaque (Anprotec, AC-AF-0018), PBMCs were washed twice with PBSdef to remove thrombocytes. A hypotonic lysis of erythrocytes was performed for 40 seconds using 0.2 % (w/v) NaCl. After washing, the cells were resuspended in RPMI (Pan Biotech, P04-18000) supplemented with 100 IU ml^-1^ penicillin, 100 µg ml^-1^ streptomycin (pen/strep) and 0,1 % BSA. For enrichment of monocytes, a counter flow centrifugation protocol was established as described in (43). Elutriated monocytes were cultured in RPMI supplemented with 2 mM glutamine, pen/strep, 1 % non-essential amino acids, 1 mM sodium pyruvate, 2 % human AB-serum. Madin-Darby canine kidney cells (MDCK) and Chinese hamster ovary cells (CHO) were cultured in Dulbecco’s modified eagle medium (DMEM, Pan Biotech, P04-03600) supplemented with 10 % FCS, pen/strep, 2 mM glutamine and 0.05 mM β-mercaptoethanol.

### Negative Selection of PBMC Subpopulations Using MACS Separation System

Untouched subpopulations of PBMCs were isolated with MACS negative isolation kits according to manufacturer’s protocol: Miltenyi, 130-096-535 (isolation of CD3^+^ cells), Miltenyi, 130-091-151 (isolation of CD20^+^ cells), Miltenyi, 130-117-337 (isolation of CD14^+^ cells) and Miltenyi, 130-097-415 (isolation of CD303^+^ cells).

### Recombinant Viruses

Recombinant influenza A viruses were generated and characterized previously (27, 40, 41). They shared six internal genes of the laboratory strain A/Puerto Rico/8/1934 (PR8) (H1N1) and contained the HA and NA genes of a seasonal human virus A/Memphis/14/1996 (H1N1) (Mem), pandemic virus A/Hong Kong/1/1968 (H3N2) (HK), zoonotic avian virus A/Vietnam/1203/2004 (H5N1) (VN) and HA mutants of HK and VN (HK-H17R, HK-R2 and VN-K58I) (see **Table 1**). The HA of the H5N1 viruses contained a deletion of the polybasic cleavage site introduced by mutagenesis, which allowed their use under BSL2 conditions. All viruses were propagated in MDCK cells, purified *via* ultracentrifugation through 20 % sucrose cushion, re-suspended in DMEM supplemented with 0.1 % BSA and stored in aliquots at -80°C. In some experiments, the viruses were buffered by addition of sodium bicarbonate (0.15 % v/v) and inactivated with beta-propiolactone (BPL, 0.05 % v/v) for 3-days incubation at 4°C.

**Table 1:** Recombinant PR8-based IAVs used in this study^a^.

Abbrev.	Source of HA and NA	Host species	Receptor-binding specificity^b^	pH of conformational transition^c^
**HK**	A/Hong Kong/1/1968 (H3N2)	Human (pandemic)	2-6 ***>*** 2-3	4.9
**HK-H17R**	H17R mutation in HA of HK	Human (pandemic)	2-6 ***>*** 2-3	5.3
**HK-R2**	L226Q+S228G mutation in HA of HK	Human (pandemic)	2-3	4.8
**Mem**	A/Memphis/14/1996 (H1N1)	Human (seasonal)	2-6	4.7
**VN**	A/Vietnam/1203/2004 (H5N1)	Poultry (zoonotic)	2-3	5.7
**VN-K58I**	K58I mutation in HA of VN	Poultry (zoonotic)	2-3	5.1

a All viruses share six gene segments of the laboratory strain A/Puerto Rico/1934 and differ by the source of HA and NA. The viruses were generated and their receptor specificity and conformational stability were characterized as described in previous publications (27, 41, 42, 44).

b Ability of the virus to bind either Neu5Ac2-6Gal-teminated or Neu5Ac2-3Gal-terminated sialylglycopolymers or both receptor types. The symbol > indicates stronger binding to the receptor type indicated.

c pH values at which 50% of the HA undergo acid-induced conformational transition and become sensitive to proteolytic degradation. These values correlate with the viral pH optimum of fusion and inversely correlate with conformational stability of HA.

### Virus Titration

Hemagglutination titers of the viruses were determined using 1% chicken red blood cells in accord with standard procedure (44). Viral infectious titers were determined using single-cycle focus formation assay in MDCK cells as described previously (42). In brief, MDCK cells in 96-well plates were infected with 0.1 ml of serial 10-fold dilutions of viruses in DMEM containing 0.1 % BSA, pen/strep and 2 mM glutamine. No trypsin was added to the medium to avoid viral multicycle replication. The cultures were incubated overnight, fixed with 4 % paraformaldehyde (PFA), and immune-stained for viral NP. Numbers of infected cells per well were counted under the microscope for the virus dilution that produced from 30 to 300 infected cells per well and recalculated into numbers of focus-forming units (FFU) per ml of the original undiluted virus suspensions.

### Biotinylated IAVs

Wild type IAVs A/Memphis/14/1996 (H1N1) (Mem-H1N1) and A/Mallard/Alberta/119/1998 (H1N1) (Mal-H1N1) were provided by Robert Webster, St. Jude Children’s Research Hospital, Memphis, TN, USA. The viruses were amplified in MDCK cells, pelleted by ultracentrifugation through 20 % sucrose cushion, and the pellets were re-suspended in PBSdef. The viruses were labelled with EZ-Link Sulfo-NHS-LC-Biotin (Thermo Scientific, No. 21335) in accord with the manufacturer’s instructions. In brief, we added 30 μl of the 10-mM solution of the biotinylation reagent to 1 ml of the suspension in PBSdef containing 5 mg of the total viral protein and incubated the mixture overnight at 4°C. The labelled virus was purified from the non-conjugated reagent by pelleting through 20 % sucrose. Pelleted virus was re-suspended in 2.5 ml of PBSdef containing 0.1 % BSA and was stored in aliquots at -80°C.

### Infection of PBMCs

Infection of primary human PBMCs, isolated subpopulations of PBMCs and PBMCs-derived macrophages were carried out at a cell density of 5 x 10^6^ cells/ml in RPMI supplemented with 0. 1% BSA, 1 % glutamine and pen/strep using individual Teflon vials with rounded interior (Savillex, 200-015-20). Recombinant influenza viruses were added at multiplicity of infection (MOI) 3, 0.3 and 0.03 FFU/cell. hIL-3 (Peprotech, #200-03-100UG) was added in a final concentration of 10 ng/ml. Infected cells were incubated for 6-8 h at 37°C, 5 % CO_2_. In these experiments, we studied the ability of the viruses to enter the cells and to initiate synthesis of viral proteins as measured by the detection of NP production *via* flow cytometry.

### Flow Cytometry

The cells in Teflon vials were incubated in an ice-cold water bath for 15 min, re-suspended by pipetting and fixed in 2 % PFA for 20 min at room temperature (RT). Fixed cells were washed twice by pelleting at 540 x g for 3 min and re-suspended in FACS buffer (3 % FCS, 2 mM EDTA, 0.01 % sodium azide in PBSdef). The cells were permeabilized by incubation for 30 min at RT in saponin buffer (0.5 % saponin, 3 % FCS, 2 mM EDTA, 0.01 % sodium azide in PBSdef) and washed twice with FACS buffer. Infected cells were detected by immune-staining with monoclonal antibody against viral NP protein (Abcam, ab43821) and Alexa633-labelled anti-rabbit antibodies (Invitrogen, A21052). Cell type specific surface markers were immune-stained using the following fluorescently-labelled antibodies according to manufacturer’s protocols: anti-CD3-FITC (BD Pharmingen, 555332), anti-CD4-FITC (BD Pharmingen, 555346), anti-CD8-PE (BD Bioscience, 340046), anti-CD20-FITC (Miltenyi Biotec, 130-111-337), anti-CD56-FITC (BioLegend, 318303), anti-CD14-PE (BD Pharmingen, 555399), anti-CD303-FITC (Miltenyi Biotec, 130-113-192), anti-CD1c-FITC (Miltenyi Biotec, 120-000-888). All incubations with antibodies were performed for 45 min at 4°C. Flow cytometric analysis was done using BD FACSCalibur with Cell Quest Pro V5.2 for collection and FlowJo v10.0 for analyzing of the data.

### Sialic Acid Expression

Cell surface expression of sialic acids was studied using biotinylated plant lectins and biotinylated influenza viruses, which preferentially bound to either Neu5Ac2-3Gal or Neu5Ac2-6Gal motifs. MDCK and CHO cells were detached by 1 x trypsin solution containing 0,02 % EDTA in PBSdef and kept on ice. PBMCs were prepared as described above. Cells were counted, diluted to 1 x 10^6^ cells per FACS tube, and washed once with PBSdef. For staining with plant lectins, the cells were incubated for 1 h at room temperature with biotinylated *Sambucus nigra* lectin (SNA) (Vector Labs, B-1305, final concentration 0,4 µg/ml) or *Maackia amurensis* lectin (MAL I) (Vector Labs, M-1315, final concentration 1 µg/ml) and washed twice with the lectin buffer (0.01 mM MnCl_2_, 0.1 mM CaCl_2_, 0.1 mM MgCl_2_, 0.1 % BSA in PBSdef). For staining with IAVs, biotinylated viruses Mem-H1N1 (final concentration of 200 µg/ml) and Mal-H1N1 (final concentration of 10 µg/ml) were incubated with the cells for 1 h at 4°C and washed twice with the FACS buffer. The cells were fixed in 2% PFA for 20 min at RT, washed twice again with either lectin buffer or FACS buffer and incubated with APC-labelled streptavidin diluted 1:2000 in lectin buffer for 40 min at 4°C. In the case of PBMCs, the staining for cell type specific surface markers was performed prior to lectin and virus binding as described earlier. Finally, 20000 events per sample were acquired by flow cytometry (FACSCalibur) with Cell Quest Pro V5.2 for data collection. The mean fluorescence intensity (MFI) was calculated for each sample using FlowJo v10.0.

### Assessment of the Effect of Extracellular pH on IAV Infectivity

MDCK cells were seeded in DMEM (Pan Biotech, P04-03600) supplemented with 10 % FCS, pen/strep, 2 mM glutamine and 0.05 mM β-mercaptoethanol in 48well-plates. On the next day, cells were washed twice with PBS containing Mg^2+^, Ca^2+^ (Pan Biotech, P04-35500), followed by addition of RPMI supplemented with pen/strep, 0.1 % BSA and adjusted to the indicated pH values (between 8.0 and 5.0) with either HCl or NaOH. After 8 hours, flow cytometry was used to determine the intracellular expression of viral NP. The same procedure was adapted for PBMC, which were isolated and counted as described above.

### Western Blotting

To obtain protein lysates of individual cell subpopulations, 17 x 10^6^ PBMCs were stained for specific surface markers as described above and subjected to cell sorting (BD FACS Aria III, Core facility flow cytometry). At least 1 x 10^5^ cells were sorted and lysed in 10 µl PBSdef supplemented with 0.1% Triton-X-100, 5 mM EDTA, and HALT-protease inhibitor cocktail (ThermoScientidic, #1862209) for 40 min at 4 °C. Nuclei were pelleted at 14.000 x g for 10 min. The supernatant was transferred to a new tube and supplemented with sample dye buffer containing SDS and β-mercaptoethanol before heating for 5 min at 95°C. The material was separated using precast 4-20% SDS-PAGE gels (Abcam, ab119205) with Run Blue running buffer (Abcam, ab119197) followed by blotting on nitrocellulose membrane (Ge Healthcare, 10600017) by semi dry-blotting according to the manufacturer’s instructions. IFITM3 and actin were detected by using rabbit anti-IFITM3 (Abcam, 15592) and mouse monoclonal anti-actin (Sigma, A2228-100UL), peroxidase coupled secondary antibodies anti-rabbit (Jackson Immuno Research, 111-035-045), anti-mouse (Jackson Immuno Research, 115-035-062) and Femto chemiluminescence peroxidase substrate (ThermoScientific, 34094). The immune-stained blots were analyzed using a ChemiDoc reader with ImageLab software (5.2) (BioRad).

### siRNA Transfection

Elutriated monocytes were treated following the instruction manual of Amaxa human monocyte nucleofector kit (Lonza, VPA-1007). In brief, elutriated monocytes were washed once with 0.1 % BSA in PBSdef and resuspended at 8 x 10^6^ cells/100 µl in supplemented nucleofector solution. 100 pmol of unspecific control scRNA (Ambion Life technologies, 4390843) or siRNA specific for IFITM3 (Ambion Life technologies, s195035) were used for electroporation. After incubation for 2 days in RPMI (Pan Biotech, P04-18000) supplemented with 20 ng/ml human recombinant GM-CSF (PeproTech, 300-03-5UG) at 37°C, cells were washed with PBSdef and infected with recombinant IAVs according to the infection protocol described above.

### Detection of Cytokines in Human pDCs

Human IFN-α and interleukin 6 (IL-6) were quantified by ELISA 24 hours after infection. The following antibodies were used: capture: rat anti-human IL-6 (Pharmingen, 554543) or anti-human IFN-α coating antibody (Bender, 2010-10), detection: biotin rat anti-human IL-6 (Pharmingen, 554546) followed by streptavidin-POD conjugate (Roche, 11089153001) or anti-human-IFN-α HRP-conjugate (eBioscience, BM216MSTK). As standards, recombinant human IL-6 (Peprotech, 200-06) or human IFN-α (Peprotech, 300-02A) were used. HRP-substrate solution contained 1 mg/ml OPD (Sigma, P7288) and 0.03% hydrogen peroxide in substrate buffer (38 mM citric acid, 66 mM disodium hydrogen phosphate). Synthetic DNA oligonucleotide with a CpG motive (CpG 2216) (TIB Molbiol) was used as control for TLR activation. Absorbance values were detected with EMax-plate photometer (Molecular Devices) with SoftMaxPro V5-Sofware.

### RNA Isolation and qRT-PCR

Total cellular RNA from 5x10^5^ human PBMCs was isolated using Extrazol (Blirt S.A.,EM30-100) according to the manufacturer’s protocol. The quality of RNA was assayed using Nano Drop spectrophotometer, and cDNA was synthesized from 500 ng RNA using QuantiTect^®^ Reverse Transcription Kit (Qiagen, 205310). qRT-PCR was performed with a BioRads MJMini-Cycler using PowerUP SYBR Green Master Mix (Appliedbiosystems, A25741) and specific primers [Qantitect Primer Assay (Qiagen, 249900)] for the following target genes *RPS11* (QT00061510), *IFN-α* (QT00201964), *IFN-β* (QT00203763), *IL-6* (QT00083720), *CCL5* (QT00090083) and *RIG-I* (DDX58(QT00040509). PCR temperature profile was 95°C for 5 min, followed by 45 cycles of 94°C for 5 sec, 55°C for 30 sec and 72°C for 20 sec. The final extension was done at 72°C for 5 min, followed by melting curve from 60°C to 95°C with increments of 0.5°C every 10 sec. All the reactions were performed in duplicates. Cytokine expression was normalized to the endogenous housekeeping control RNA *RPS11* and calculated according 2^–ΔΔCt^ method. The normalized relative expression was calculated as the percentage of HK virus values for each donor.

### Statistics

Statistical analyses were performed using GraphPad Prism 9.0. If not indicated otherwise, the figures show data from at least three individual biological replicates. The bars or horizontal lines indicate the group means. As degree of dispersion, the standard deviation was calculated and presented as length of error bar. If not stated in the individual figures, the one-way ANOVA followed by Dunnett’s or Turkey’s multiple comparison tests were used to compare multiple groups. Asterisks depict p values as follows: *p<0.05, **p<0.01, ***p<0.001, ****p<0.0001.

## Results

### Recombinant Viruses

**Figure 1:**
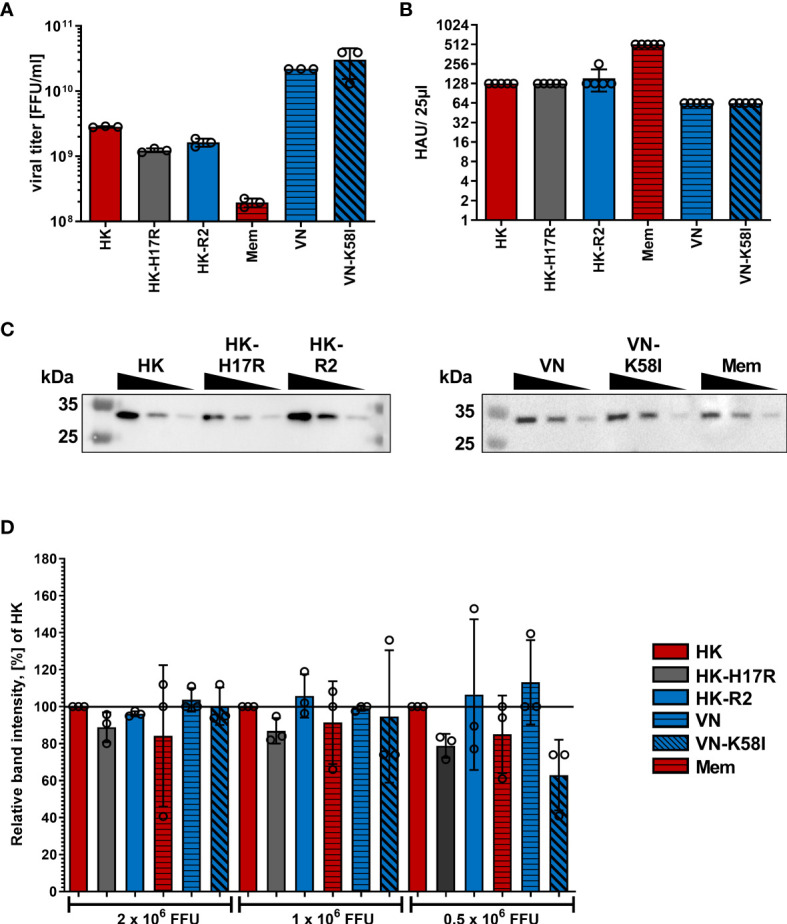
Characterization of virus stocks. **(A)** Viral titers in focus-forming units (FFU) per ml were determined using focus-formation assay in MDCK cells. Mean data of 3 independent experiments, each performed in triplicates are shown. **(B)** Hemagglutination titers with chicken red blood cells of viral suspensions diluted to viral infectious titers 1x10^7^ FFU/ml. Mean HAU of 5 independent experiments are shown. P values were determined by ANOVA followed by Turkey’s multiple comparison test and shown if significant. **(C)** Exemplary western blot for IAV M1 protein using samples containing 2x10^6^, 1x10^6^ and 0.5x10^6^ FFU of the indicated viruses. **(D)** Quantification of viral M1 protein after Western blotting. Band intensity of the M1 protein was normalized to HK in each dilution. Mean data of 3 independent western blots are shown. Significance determined by ANOVA followed by Dunnett’s multiple comparison test compared to HK within each FFU of virus. P values shown if significant.

To characterize how receptor specificity and membrane fusion activity of the HA affects tropism of IAVs to human immune cells and response of the cells to IAV infection we used a panel of three 2:6 recombinant IAVs that shared six gene segments of PR8 and that contained the HAs of A/Vietnam/1203/2004 (H5N1), A/Hong Kong/1/1968 (H3N2), and A/Memphis/14/1996 (H1N1), as representative HAs of zoonotic avian, pandemic and seasonal IAVs infecting humans, respectively. The previous characterization of these viruses is summarized in **Table 1** (27, 40–42). Moreover, to separately study effects of receptor-binding specificity and conformational stability of IAVs and to exclude potential effects of the non-homologous NAs, we used three IAVs containing point mutations in the functional regions of the HA responsible for either receptor specificity (HK-R2) or stability (HK-H17R and VN-K58I) (27, 40–42, 45, 46). All viral stocks showed comparable characteristics such as virus titers in focus-formation and hemagglutination assays as well as M1 protein content (**Figure 1**).

### Receptor Specificity Affects IAV Infection in PBMCs

**Figure 2:**
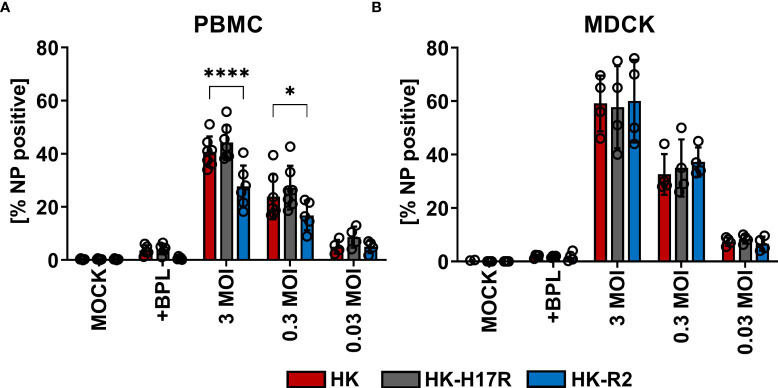
Infection of PBMCs with HK and its HA variants HK-H17R and HK-R2. **(A)** Human PBMCs and **(B)** MDCK cells were incubated with IAVs at MOIs of 3, 0.3 and 0.03 for 8 hours. FACS analysis was used to determine the percentage of NP positive cells. Uninfected cells (MOCK) and cells inoculated with BPL-inactivated viruses at the dose corresponding to MOI 3 were used as controls. The mean of NP-positive cells ± SD are shown for at least 4 independent donors. Significance determined by ANOVA followed by Dunnett’s multiple comparison test compared to HK. P values shown if significant. *p < 0.05, ****p < 0.0001.

**Figure 3:**
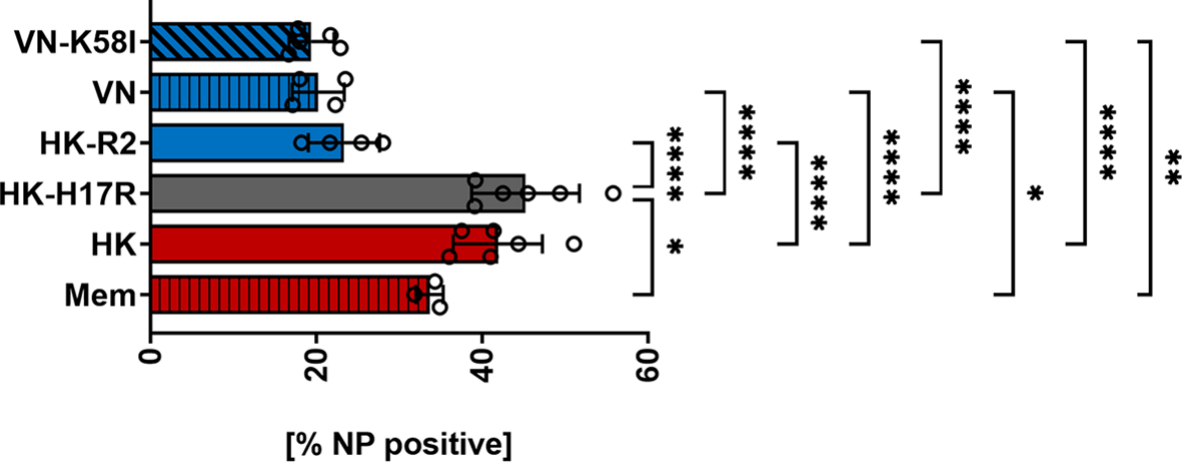
Infection of PBMCs with IAV differing in HA. Primary human PBMCs were infected with 3 MOI of the seasonal human (Mem), pandemic human (HK, HK-H17R) and avian viruses (VN, VN-K58I, HK-R2) for 8 hours. FACS analysis was used to determine the percentage of NP positive cells. The mean of NP-positive cells ± SD are shown for at least 3 independent donors. Significance determined by ANOVA followed by Turkey’s multiple comparison test. P values shown if significant *p < 0.05, **p < 0.01, ****p < 0.0001.

First, we compared HK and its point mutants differing by receptor specificity (HK-R2) and conformational stability (HK-H17R) for their infectivity for human immune cells. In a single infection cycle approach primary human PBMCs were inoculated at three different MOI (based on viral infectious titers determined in MDCK cells), and infected cells were identified by their intracellular expression of NP protein using flow cytometry (**Figure 2A**). Cells inoculated with BPL-inactivated viruses were used as mock-infected control. HK and HK-H17R infected PBMCs at similar levels, whereas HK-R2 was significantly less infectious than HK at MOI 3 and 0.3 (**Figure 2A**). In contrast to PBMCs, MDCK cells, which are readily susceptible to both human and avian viruses, were equally well infected by all 3 viruses (**Figure 2B**). To confirm observed reduced infection of PBMCs by IAVs with avian-type receptor specificity we infected PBMCs with 3 additional viruses, Mem, VN and a fusion mutant of VN (VN-K58I). Indeed, the infection levels of PBMCs with VN (avian-type receptor specificity) were significantly lower when compared to Mem and HK (human-type receptor specificity) (**Figure 3**). Again, the differences in HA conformational stability (VN versus VN-K58I and HK versus HK-H17R) did not result in significant differences in infection.

**Figure 4:**
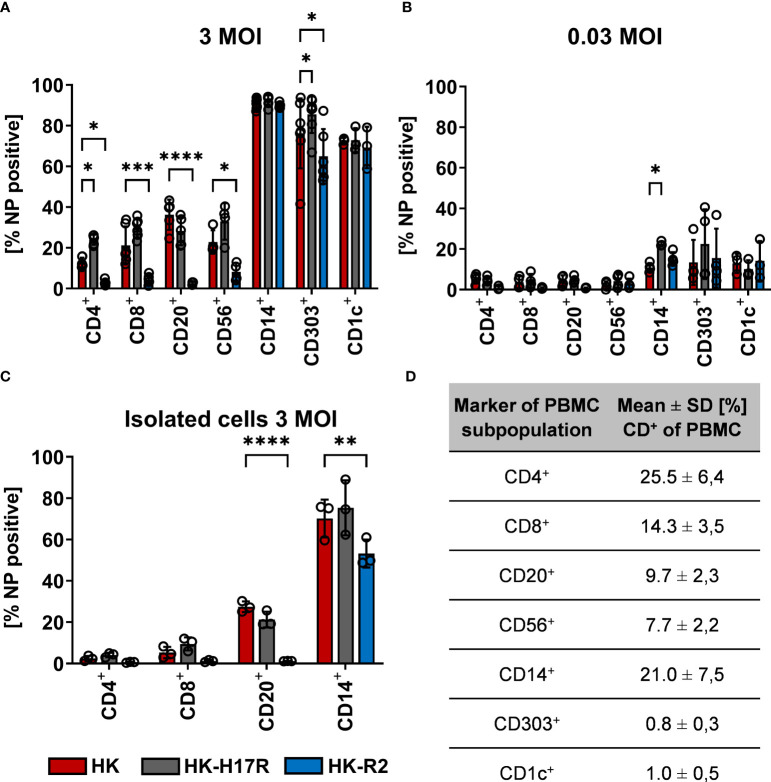
Influence of receptor specificity and conformational stability on the infection pattern in subpopulations of human PBMCs. **(A, B)** Human PBMCs or **(C)** MACS-isolated subpopulations were incubated for 8 hours with indicated viruses at MOI 3 **(A, C)** or MOI 0.03 **(B)**. Flow cytometric analysis was used to determine the intracellular expression of viral NP and the expression of cell type specific CD-molecules (CD4^+^, T helper cells; CD8^+^, cytotoxic T cells; CD20^+^, B cells; CD56^+^, NK cells; CD14^+^, monocytes; CD303^+^, plasmacytoid dendritic cells; CD1c^+^, myeloid dendritic cells). The mean values of NP-positive cells ± SD are shown for at least 3 independent donors. Significance determined by ANOVA followed by Dunnett’s multiple comparison test compared to HK. P values shown if significant *p < 0.05, **p < 0.01, ***p < 0.001, ****p < 0.0001. **(D)** Mean ± SD [%] of CD-marker positive cells of each subpopulation of PBMCs after 8 hours of infection. Data from at least 3 independent donors are shown.

Further characterizing the role of receptor specificity and conformational stability in infection of specific immune cells within the PBMC mixture, we double-stained inoculated cells for virus infection and cellular surface markers (**Table 2**). In general, monocytes, myeloid (mDCs) and plasmacytoid dendritic cells (pDC) were efficiently infected (80 to 90 %) with HK, HK-H17R, and HK-R2, whereas infection levels in lymphocytes and NK cells were below 40 % (**Figure 4A**). Notably, the patterns of infection varied depending on both cell type and the virus strain. Thus, lymphocytes (CD4^+^ and CD8^+^ T cells and CD20^+^ B cells) and NK cells (CD56^+^) were almost completely resistant to HK-R2, whereas HK and HK-H17R infected these cells at a level of 20 to 40 % (MOI 3) with HK-H17R showing a higher infection rate than HK in CD4^+^ T cells and NK cells. (**Figure 4A**). In contrast, CD14^+^ monocytes were infected by HK, HK-R2 and HK-H17R with 90 % efficiency (MOI 3). However, at MOI 0.03 infection rate by HK-H17R was higher when compared to HK and HK-R2 (**Figure 4B**). For CD1c^+^ mDCs an equal infectivity of all 3 viruses was observed, whereas CD303^+^ pDCs cells were differentially infected (HK-H17R > HK > HK-R2) at 3 MOIs used (**Figure 4A**). Even though not statistically significant, the infection pattern of CD1c^+^ mDCs and CD303^+^ pDCs remains constant in infection with 0.03 MOI (**Figure 4B**).

**Table 2:** Cell type specific surface marker for human PBMC subpopulation.

Cell type	Marker
Lymphocytes	CD3^+^
T_H_ cells	CD4^+^
T_C_ cells	CD8^+^
B cells	CD20^+^
NK cells	CD56^+^
Classical monocytes	CD14^+^
Plasmacytoid DCs	CD303^+^
Myeloid DCs	CD1c^+^

To assess susceptibility to viruses of individual cell types in the absence of other types of cells present in PBMCs we studied cell subpopulations purified by MACS before infection. We decided to isolate both T-cell subpopulations CD4^+^ and CD8^+^ as well as CD20^+^ B-cells and CD14^+^ monocytes, since interaction of these cells was hypothesized earlier to be relevant for infection (37, 38) (also see *Discussion*). Isolation of CD303^+^ pDCs was omitted due to technical limitation in purifying sufficient amounts of cells for infection studies. Although the rates of infection by all viruses were markedly reduced, the pattern of infection of the isolated immune cell types agreed with their pattern of infection within the PBMC mixture (**Figure 4C**). Interestingly, no infection in CD20^+^ B-cells with HK-R2 could be detected, while the infection rate of IAVs with the human receptor specificity was still detectable at rates between 27 % (HK) and 21 % (HK-H17R). In summary, IAV infection of lymphocytes and NK cells strongly depended on HA receptor specificity, the avian-type virus being markedly less infectious for these cells than human-type viruses. In contrast, infection of monocytes and pDCs correlated with conformational stability of the viral HA, the virus fusing at a higher pH being more infectious.

### Expression of Sialic Acids and Binding of the Viruses to Cells

**Figure 5:**
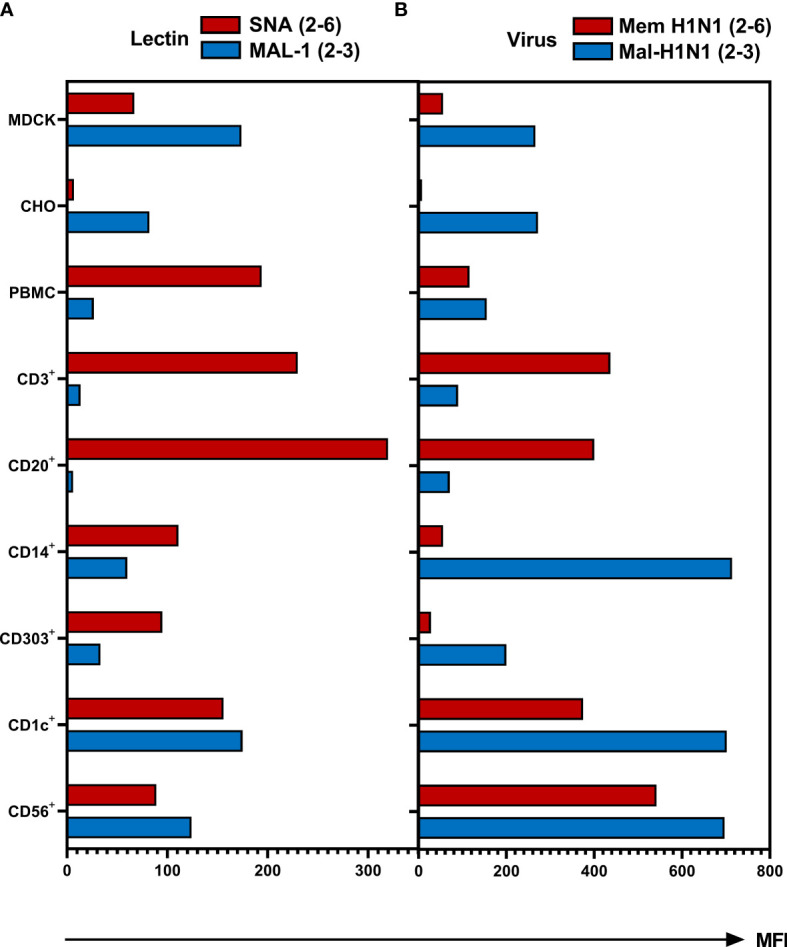
Binding of biotinylated lectins and viruses to human PBMCs. The MDCK and CHO cells and PBMCs were incubated for 1 hour with **(A)** biotinylated *Sambucus nigra* lectin (SNA, final concentration 0.4 µg/ml) or biotinylated *Maackia amurensis* lectin 1 (MAL-1, final concentration 1 µg/ml) at room temperature or **(B)** with biotinylated IAVs Mem-H1N1(final concentration 200 µg/ml) or Mal-H1N1 (final concentration 10 µg/ml) at 4 °C. Lectin or virus binding was detected as mean fluorescence intensity (MFI) after staining with streptavidin–APC. Cells were then fixed and stained for subpopulation as described in Material and Methods and subjected to flow cytometric analysis. Data of one representative donor are shown.

To test whether observed differences in susceptibility of different types of cells to IAV infection correlate with the cell type-specific variation in the expression of sialic acid receptors, we determined expression of SA2-3Gal- and SA2-6Gal-containing glycans using biotinylated SA-binding proteins and fluorescently labelled streptavidin. Plant lectin *Sambucus nigra* agglutinin (SNA) and seasonal human IAVs Mem-H1N1 were used to detect 2-6 SA; *Maackia amurensis* lectin (MAL-1) and avian IAV Mal-H1N1 were employed for the specific detection of 2-3 SA (**Figure 5**). MDCK and CHO served as controls and as shown previously, MDCK cells express both types of SA-Gal linkages (47, 48), whereas CHO cells only express SA2-3Gal (49, 50). However, based on lectin binding (**Figure 5A**), CD14^+^ monocytes, CD303^+^ pDCs, CD1c^+^ mDCs and CD56^+^ NK cells expressed both 2-6- and 2-3-linked SA. The expression of 2-3-linked SA on CD1c^+^ mDCs and CD56^+^ NK cells was more pronounced compared to CD14^+^ monocytes and CD303^+^ pDCs. In contrast, the CD3^+^ lymphocytes and CD20^+^ B cells expressed the highest levels of 2-6-linked SA, but the expression of 2-3-linked SA on these cells was close to the detection limit of the assay. Of note, the pattern of the binding of biotinylated IAVs (**Figure 5B**) did not fully correlate with the binding of the corresponding lectins (**Figure 5A**). Virus binding reflected SA expression of 2-3 SA on CHO cells as well as the more pronounced abundance of the 2-6 SA on CD3^+^ and CD20^+^ cells. Similarly to CHO, the expression of both SA variants on PBMCs, CD1c^+^, CD56^+^ and MDCK cells was recapitulated by virus binding. However, the pattern of SA expression on CD14^+^ and CD303^+^ cell subpopulations detected by lectins did not correlate with the pattern of the virus binding. This apparent discrepancy can be related to the differences in the binding specificity of the plant lectins and IAV HA, in particular, to the ability of the sialic acid binding plant and viral proteins to recognize oligosaccharide parts of the sialoglycans penultimate to the terminal SA-Gal moieties (51–53). Furthermore, efficient binding of both viruses by monocytes and pDCs may be explained by sialic-independent binding mechanisms (see *Discussion*). In summary, innate immune cells express both 2-6- and 2-3-linked SA, whereas lymphocytes mainly express 2-6-linked SA, this pattern correlates with relatively inefficient binding of the avian virus to lymphocytes.

### Effect of Extracellular pH on IAV Infectivity for PBMCs

**Figure 6:**
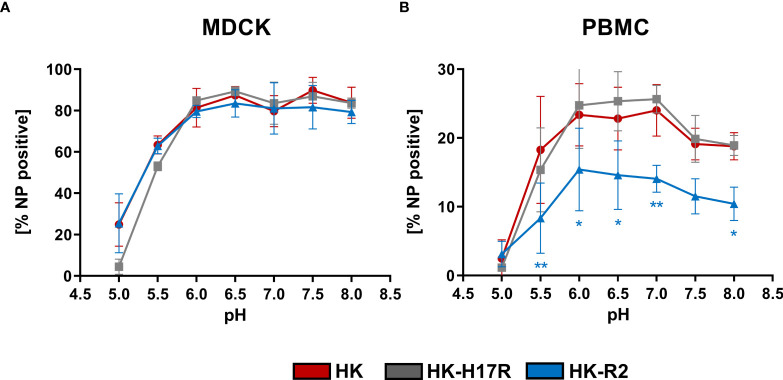
Effect of extracellular pH on infection in **(A)** MDCK cells and **(B)** human PBMCs. The MDCK cells and PBMCs were incubated with IAVs at MOI 3 and 0.3, respectively, at different pH for 8 hours. Flow cytometric analysis was used to determine the intracellular expression of viral NP. The mean of NP positive cells ± SD are shown for 3 independent donors and MDCK infections. Significance determined by ANOVA followed by Dunnett’s multiple comparison test compared to HK. P values shown if significant *p < 0.05, **p < 0.01.

IAVs enter the human body *via* oral and nasal cavities and replicate in the human respiratory tract. The pH of the human respiratory tract can vary from pH 6.3 in the nasal cavity to pH 7.5 in the lung (16). In line with this, it was speculated that the interplay between HA conformational stability and extracellular pH in the host target tissues can affect infectivity of IAVs (54) and act as a selection mechanism (55). Therefore, we tested whether infectivity of IAVs for PBMCs is affected by changes in extracellular pH. PBMCs and MDCK cells were transferred and incubated in the RPMI-based infection medium adjusted to pH in the range from 8.0 to 5.0. Infection with IAVs HK, HK-R2, and HK-H17R was performed for 8 h (**Figure 6**). The infection efficiency in MDCK with all 3 viruses remained constant at 80 % in a pH range from 8.0 to 6.0 and decreased to 60 % at pH 5.5 (**Figure 6A**). At pH 5.0 HK and HK-R2 showed a similar infection rate of 25 %, while the infectivity of HK-H17R with the less stable HA dropped to 4 % due to irreversible pH-triggered conformational transition and inactivation of the HA molecule (**Figure 6A**). We did not determine whether direct fusion of the viruses with plasma membranes of the cells contributed to viral infection under these conditions. Somewhat different patterns of IAV infection was observed under the same conditions in PBMCs (**Figure 6B**). HK-R2 showed generally reduced infection rate compared to the HK and HK-H17R. The infectivity of all three viruses peaked at pH between 7 and 6 and decreased above pH 7 and below pH 6. We concluded that the differences in HA conformational stability of the IAVs studied have no effect on the pattern of their infectivity for PBMCs at different extracellular pH.

### IFITM3 Is a Major Restriction Factor for IAVs With High Conformational Stability in Human Primary Macrophages

**Figure 7:**
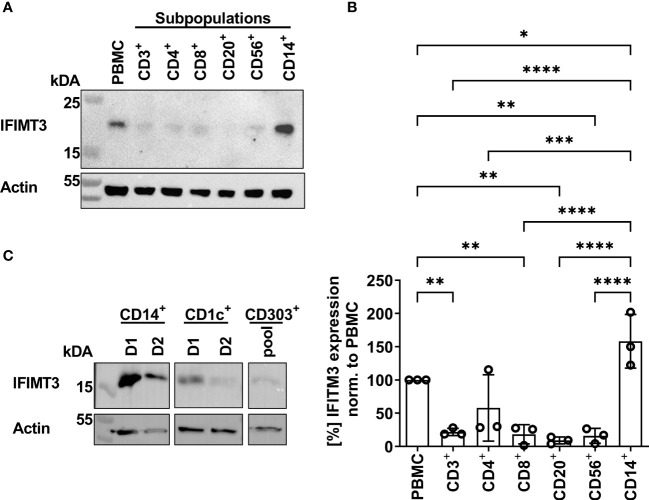
Detection of IFITM3 expression in PBMC subpopulations. **(A)** Western blot for IFITM3 (upper part) and actin (lower part) using 200.000 cells/lane of either total PBMCs or sorted subpopulations of PBMCs (representative data for the cells from one donor). **(B)** Quantification of three WB from independent donors for basal IFITM3 expression in PBMCs and subpopulations as in **(A)**. Intensity values of western blot analysis were normalized to non-fractionated PBMCs value and expressed as mean [%] ± SD. Significance determined by ANOVA followed by Newmann-Keuls multiple comparison test. P values shown if significant *p < 0.05, **p < 0.01, ***p < 0.001, ****p < 0.0001. **(C)** Western blot analysis of IFITM3 in myeloid cells isolated by FACS. 100.000 cells/lane of monocytes (CD14^+^), mDCs (CD1c^+^) of two individual donors and a pooled lysate of pDCs (CD303^+^) from these donors were used for detection of IFITM3 (upper panel) and actin (lower panel).

**Figure 8:**
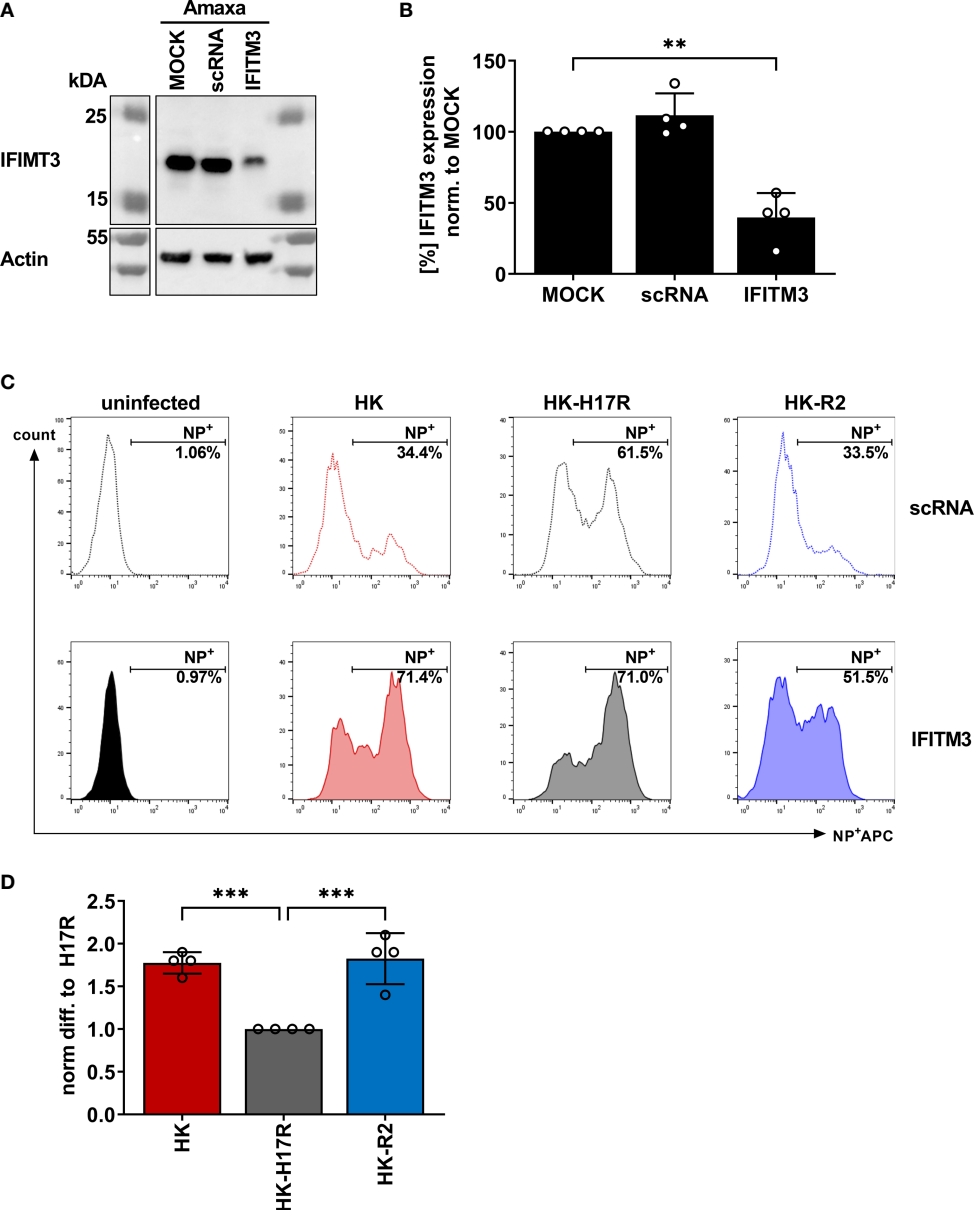
Assessment of IAV infection in primary human macrophages after siRNA-mediated IFITM3 knock down. **(A)** Representative western blot for IFITM3 (upper part) and actin (lower part) using elutriated, GM-CSF differentiated macrophages mock-treated or treated with scrambled or IFITM3-specific siRNA. **(B)** Quantification of IFITM3 knock down normalized to MOCK using 4 independent experiments. Significance determined by ANOVA followed by Dunnett’s multiple comparison test. P values shown if significant **p< 0.01. **(C)** Detection of intracellular NP by FACS 8 hours post infection in scRNA or specific IFITM3 siRNA treated primary human macrophages infected with the indicated viruses at MOI 0.3. **(D)** Fold differences in virus infected cells (NP+) normalized to the scRNA-sample using cells from 4 different donors. Significance determined by ANOVA followed by Dunnett’s multiple comparison test. P values shown if significant ***p < 0.001.

In previous studies we found that sensitivity of IAVs to the antiviral protein IFITM3 (interferon induced transmembrane protein 3) was dependent on the stability of the HA and that this effect contributed to differences in viral cell tropism especially in primary human microvascular endothelial cells (27, 40). As IFITM3 expression in various human immune cells has not been studied in detail, we analyzed the basal expression and distribution of IFITM3 in total human PBMCs and PBMC-derived immune cell subtypes. Western blot analysis of PBMCs and sorted subpopulations demonstrated various levels of IFITM3 expression (**Figures 7A, B**). The CD14^+^ monocytes represented that major source of IFITM3 in PBMCs, the T-lymphocytes and NK cells expressed much smaller amounts compared to monocytes, whereas CD20^+^ B cells showed from very weak to undetectable IFITM3 expression (**Figures 7A, B**). Since the results demonstrated differences between the lymphoid and myeloid compartment in respect to IFITM3 expression, we characterized additional subpopulations such as CD1c^+^ mDCs and pDCs (CD303^+^). Interestingly, IFITM3 could be readily detected in CD1c^+^ DCs but was expressed much weaker in CD303^+^ pDCs (**Figure 7C**). To further investigate the role of IFITM3 in viral restriction and IFITM3 interplay with HA stability, we isolated CD14^+^ monocytes, performed siRNA mediated knock down of IFITM3 and infected these cells with HK, HK-R2, and HK-H17R. IFITM3 expression was reduced by at least 50 % in monocytes after specific siRNA treatment (**Figures 8A, B**). A scrambled siRNA had no effect on IFITM3 expression when compared to MOCK-treated cells (**Figures 8A, B**). In scrambled siRNA-treated monocytes HK and HK-R2 were stronger restricted when compared to HK-H17R (**Figure 8C**). In contrast, monocytes treated with an IFITM3-specific siRNA showed a loss of viral restriction, since HK and HK-R2 showed higher infection rates and reached the infectivity of HK-H17R (**Figure 8C**). To quantify differences in the infection rate of all three viruses, we calculated percentages of infected cells in siRNA-treated monocytes with respect to infected scRNA-treated monocytes from four donors and normalized the results to the HK-H17R-mutant (**Figure 8D**). Viruses with the conformationally stable HA (HK and HK-R2) showed about twofold reduction of infectivity in mock-treated cells, while less stable HK-H17R showed no differences in infectivity. Collectively, these results indicate that monocytes and CD1c^+^ mDC cells express markedly higher levels of endogenous IFITM3 than any other cells subpopulation present in PBMCs. Furthermore, we confirm and extend the previous observations (27, 40) that viruses with conformationally stable HA are more effectively restricted by IFITM3 than viruses with less stable HA.

### Antiviral Response to Human and Avian-Like Viruses

**Figure 9:**
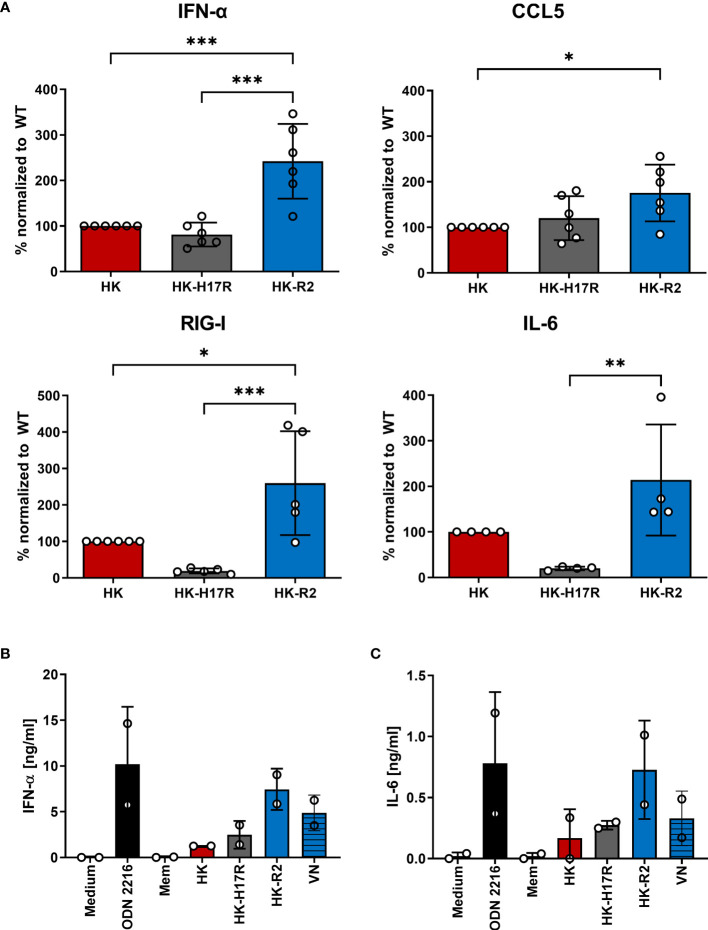
Regulation of cytokines in primary human PBMCs during infection with HK and its HA variants. **(A)** mRNA expression of cytokines (*IFN-α, CCL5, IL-6*) and nucleic acid sensor (*RIG-I*) of PBMCs inoculated at MOI 3 with HK and its HA variants HK-H17R and HK-R2 measured 6 hours p.i. using RT-PCR. Mean expression values +SD of four donors in duplicates are shown. Raw data of cytokines were normalized to the expression of the housekeeping gene RPS11 and related to HK infected samples. Significance determined by ANOVA followed by Turkey’s multiple comparison test. P values shown if significant *p < 0.05, **p < 0.01, ***p < 0.001. **(B, C)** MACS-isolated pDCs were either inoculated with recombinant IAVs at MOI 1 or stimulated with synthetic TLR-ligand ODN 2216 for 24 hours. Concentrations of **(B)** IFN-α or **(C)** IL-6 in the cell supernatants were determined by ELISA and depicted as mean amount of cytokine [ng/ml] +SD of two donors in duplicates.

To analyze the early innate immune response of immune cells infected with HK, HK-H17R, and HK-R2 mutant, we utilized RT-PCR in infected PBMCs for the detection of cytokines (e.g. *CCL5, IFN-α, IL-6*) and the RNA sensor *DDXH58* (RIG-I). In addition, release of IFN-α and IL-6 from pDCs incubated with HK, HK-H17R, HK-R2, VN and Mem was analyzed by ELISA. The infection of human PBMCs with the avian-like receptor mutant HK-R2 was accompanied by the strongest induction of all cytokines tested. mRNA levels of all tested cytokines and the RNA sensors were increased 1.5-fold (*IL6*), 2-fold (*IFN-α, CCL5*) and 2.1-fold (*RIG-I*) (**Figure 9A**). No differences in the expression of levels of *IFN-α* and *CCL5* could be observed in cells incubated with HK and its fusion mutant HK-H17R. However, the induction of *RIG-I* and *IL-6* were significantly less induced by HK-17R as compared to HK (**Figure 9A**). pDCs that were inoculated with IAVs carrying HA with specificity for 2-3-linked SA (HK-R2, VN) demonstrated the strongest IFN-α and IL-6 production (**Figures 9B, C**). In contrast, the virus with a strict preference for 2-6-linked SA and no binding to 2-3-linked SA (Mem) induced almost no cytokines. In agreement with this pattern, HK and HK-H17R which combined a strong binding to 2-6-linked SA with a weak binding to 2-3-linked SA induced intermediate levels of IFN-α and IL-6. In contrast to observed effects of the receptor specificity, the differences in HA conformational stability did not affect induction of cytokines in human pDCs (compare HK and HK-H17R, **Figures 9B, C**). Together, our results of mRNA induction and cytokine release indicate that the IFN-α and IL-6 response in human immune cells is dependent on the receptor specificity of the IAV. Thus, IAVs with an avian-virus-like specificity for 2-3-linked SA upregulate multiple cytokines and sensors of viral infections more efficiently than human viruses with 2-6-linked SA binding counterpart.

## Discussion

IAVs infecting humans can differ by receptor binding specificity and conformational stability of their surface glycoprotein HA. Both HA characteristics were shown to affect viral tropism to epithelial cells (27, 56–58). Less is known about the infection and activation of non-epithelial cells. Many studies focus on strain-dependent differences between IAVs in their replication and activation of myeloid murine and human cells [for review see (30–34)]. However, these studies typically employed wild type IAVs and could not dissect the role in the tropism of individual viral genes and/or characteristics of individual viral proteins. Li et al. demonstrated that the internal genes of highly pathogenic H5N1 IAVs, independently of their surface glycoproteins, allowed efficient viral replication in myeloid cells, thus triggering extensive cytokine production and immunopathology in mice (59). To extend these findings and to characterize further how HA properties shape viral infection and immune cell tropism in early steps of IAVs replication cycle, we used 2:6 recombinant IAVs. These IAVs shared 6 internal gene segments of the laboratory strain PR8 and differed only by the HA and NA molecules, which originated from human pandemic (H3N2), zoonotic (H5N1) and seasonal (H1N1) viruses. In our experiments, we focused on the ability of viruses to enter the cells and to initiate synthesis of viral proteins as measured by the detection of the NP protein production. No efforts were undertaken to analyze production of complete viral particles, proteolytic activation, HA activation by cellular proteases, and release of infectious viruses from PBMCs and their subsets. These aspects of the interaction of the IAVs used with innate immune cells will need further studies.

To analyze how receptor specificity influences the tropism of IAVs to human immune cells we first investigated the infection of human PBMCs. We found that all viruses, including point HA mutants with binding specificity for 2-3-linked SA (avian-type receptor), showed significantly lower infectivity for PBMCs when compared to viruses with the human-type 2-6-linked SA receptor specificity (**Figure 3**). Co-staining of different immune cell subtypes in human PBMCs showed that the reduced infection of total PBMCs by avian-type HK-R2 was caused by reduced infection of natural killer (CD56^+^) cells and almost complete resistance of lymphocytes (CD4^+^ T cells, CD8^+^, T cells, CD20^+^ B cells). In contrast to lymphocytes and NK cells, no correlation was observed between infection rates and viral receptor specificity in classical monocytes (CD14^+^), and mDCs (CD1c^+^) and pDCs (CD303^+^) (**Figures 4A, B**). Infections with MACS-isolated subpopulations show reduced infection of lymphocytes (**Figure 4C**). The resistance of lymphocytes to IAV infection correlated with lack of SA moieties determined using SA-specific plant lectins and with poor binding of labelled IAVs (**Figures 5A, B**). Interestingly, in monocytes, pDCs and mDCs no correlation between SA expression and virus binding was observed (**Figure 5**). Specialized receptors on macrophages and DCs, the C-type lectin receptors (CLRs), can bind to N-glycans on the HA and NA of IAVs thereby mediating SA-independent viral attachment and cell entry [for a review, see (60, 61)]. We assume that this mechanism can be primarily responsible for the lack of correlation between binding of lectins and IAVs to monocytes and DCs.

Our results indicate that changes in the receptor specificity of the IAVs are sufficient to alter the profile of virus-induced cytokine response of PBMCs by enhancing expression of *IFN-α*, *IL-6*, *RIG-I*, and *CCL5* on mRNA levels (**Figure 9A**) and elevation of the levels of secreted IFN-α in human pDCs (**Figure 9B**). The chemokine CCL5 (RANTES) is mainly produced in lymphocytes and facilitates further recruitment of lymphocytes to the side of infection by increasing adhesion of leukocytes to the endothelium (62). The interferon response gene RIG-I, a main sensor for viral RNA (63), and the proinflammatory cytokine IL-6, secreted by monocytes, trigger the synthesis of acute phase proteins in the liver and leading to B cell proliferation (64). Infections caused in humans by highly pathogenic avian IAVs such as H5N1 are long known to be associated with elevated levels of cytokines (6, 65–67). Our findings extend results reported by Ramos et al. that differences in binding specificity of recombinant H5N1 virus alter the cytokine release of human DCs (29). Using isolates of pandemic H1N1 and avian H7 and H5 viruses, Lee et al. showed differences in viral binding to several PBMC subpopulations (35). Extending these observations we demonstrate that susceptibility of isolated B- and T cells to IAVs depends on the receptor binding specificity of the HA. Our results also indicate that the presence of monocytes enhance susceptibility of these cells most possibly by direct cell-to-cell interaction since infection of viruses in isolated subpopulations followed the same pattern as in the PBMC mixture, but the infection rates were strongly reduced (**Figures 4A, C**). These findings are in line with the report of Mock and colleagues, which indicated that direct interaction with monocytes and/or macrophages is required for the efficient infection of lymphocytes (37). In a more recent study the authors showed that the susceptibility of human isolated B cells to H5N1 viruses was dependent on direct cell-to-cell contact of B cells and monocytes (68). Our results support and extend these data by demonstrating that direct interaction seems to be of high importance for infection of B cells with avian IAVs that bind to 2-3-linked SA but may play a minor role in infection of these cells by human IAVs, which preferentially bind to 2-6-linked SA (**Figure 4C**, CD20^+^ column). The significance of infected lymphocytes in the establishment of viral infection in the host, immune response and pathogenicity of IAVs is currently under discussion, and further studies in the field are needed. Infection of lymphocytes by H7N9 and H5N1 was reported not to cause cell death, whereas monocytes were found to undergo rapid apoptosis (35). In contrast, Nichols et al. reported that lymphocytes in general undergo apoptosis after exposure to H1N1 viruses, with cytotoxic T cells being more affected than T helper cells (69). Recently by analyzing viral replication and particle production Castro and colleagues reported direct infection of human CD8^+^ T cells and B cells in tonsils of children and their role in host-to-host transmission in asymptomatic long-term infections (70). By using single point mutants that specifically affect the HA stability without effects on receptor-binding properties we found that destabilization of the HA molecule leads to slightly increased infection of human PBMCs (**Figure 2A**).

Consistent with the hypothesis that HA conformational stability can affect the timing of the endosomal fusion and may influence cytokine release in PBMCs, we found that destabilization of HA leads to reduced levels of mRNA for *RIG-I* and *IL6*. IFITM3 is a broadly-acting antiviral molecule (71) with known anti-influenza activity (72, 73) and therefore may shape the cytokine expression profile. We found that human lymphoid cells express very low levels of IFITM3, whereas myeloid cells namely, monocytes, mDCs and pDCs are the main producing cells for IFITM3 (**Figure 7**). In experiments with human monocytes, we demonstrated that IFITM3 expression affected susceptibility of cells to IAVs with conformationally stable HA molecules and that the impairment of infection in monocytes by IAVs with stable HA could be overcome by siRNA knock down of IFITM3 in these cells (**Figure 8**). The exact mechanism behind the IFITM3-mediated impairment of the pore formation and cell entry of vRNPs of IAVs is still not understood. It was suggested that IFITM3 is recruited to the early endosome and integrated in the endosomal membrane in a cluster-dependent manner (74). Detailed mechanisms that determine susceptibility or resistance to IFITM3 of the viruses that use the endosomal trafficking compartment for cell entry are not completely understood (71, 74, 75). Sun et al. reported that human endothelial cells express IFITM3 in steady state conditions and this shapes the infection with human and avian IAVs. The authors speculated that some IAV strains overcome the first antiviral block by early escape of the endosomal compartment or other unknown mechanisms (76). Gerlach et al. demonstrated that the pH optimum of fusion of the IAVs HA molecule determines the susceptibility of IAVs to IFITM3 (40). Hensen et al. extended these findings by testing the HA mutants of the H5N1 IAVs differing by pH optimum of fusion (27). Of note, not only IFITM expression but also the endosomal pH can influence efficient infection in different cells lines (27) such as Vero cells (77) and murine RAW264.7 cells (30).

In summary, our results extend the current understanding of the role of IAV HA on viral cell tropism (**Table 3**). We show that the HA receptor specificity critically affects IAV infection of lymphocytes, with little if any effect on the infection of monocytes and DCs. This pattern correlates with sufficient levels of both human-type and avian-type sialic acid receptors on the latter two types of cells and with the deficiency of the avian-type receptors on lymphocytes. We demonstrate that conformational stability of the HA represents an important factor affecting infectivity of IAVs for monocytes and DCs, this effect being determined by high endogenous expression in these cells of the antiviral protein IFITM3. Furthermore, we show that properties of the HA may affect activation and innate responses in non-epithelial cells. These results provide a rationale for further studies on the role of the HA properties in infection and activation of non-epithelial cells, on underlying molecular mechanisms and the role of these effects in innate and specific immune responses and pathogenesis during influenza infection.

**Table 3:** Properties of HA and target cell shaping tropism of IAVs to subpopulations of human PBMCs.

Cell type	Marker	Property of HA^a^		Property of the cell^b^	
		Receptor specificity	Conformational stability	IFITM3 expression	Receptor expression
T_H_ cells	CD4^+^	**++**	**+**	n.d.^c^	*α*2-6
T_C_ cells	CD8^+^	**++**	**+**	intermediate	*α*2-6
B cells	CD20^+^	**++**	**-**	n.d.	*α*2-6
NK cells	CD56^+^	**++**	**+**	n.d.	*α*2-6 and α2-3
Classical monocytes	CD14^+^	**-**	**++**	very high	*α*2-6 and α2-3
Plasmacytoid DCs	CD303^+^	**-**	**++**	high	*α*2-6 and α2-3
Myeloid DCs	CD1c^+^	**+**	**+**	high	*α*2-6 and α2-3

a The number of plusses reflect magnitude of the effect of HA property on infection efficiency. Minus depicts a lack of a significant effect.

b Expression of IFITM3 protein and two types of sialic acid motifs (a2-6 and a2-3).

c n.d. not detected.

## Data Availability Statement

The original contributions presented in the study are included in the article. Further inquiries can be directed to the corresponding authors.

## Ethics Statement

The local Ethics committees of Justus-Liebig-University Giessen and Philipps-University Marburg approved the use of human blood samples (buffy coats) for this study. Informed consent was obtained from all blood donors, and the experiments performed conformed to the principles of the WMA Declaration of Helsinki. The patients/participants provided their written informed consent to participate in this study.

## Author Contributions

JD performed the experiments. JD, AK, MM, and SB designed the experiments. VB helped to establish immune-staining and infection protocols. HR designed and performed cell sorting panels at BD AriaIII. JW contributed to growing and characterization of the initial virus stocks and advised on handling of the viruses. JD, MM, and SB wrote the manuscript. AK, MM, and SB conceived and supervised the study. All authors approved the submitted version of the manuscript.

## Funding

This work was funded by the Deutsche Forschungsgemeinschaft (DFG, German Research Foundation) – SFB 1021-Project B02, Project-ID 197785619 to MM and SB. Open Access funding provided by the Open Access Publication Funds of Philipps-Universität Marburg.

## Conflict of Interest

The authors declare that the research was conducted in the absence of any commercial or financial relationships that could be construed as a potential conflict of interest.

## Publisher’s Note

All claims expressed in this article are solely those of the authors and do not necessarily represent those of their affiliated organizations, or those of the publisher, the editors and the reviewers. Any product that may be evaluated in this article, or claim that may be made by its manufacturer, is not guaranteed or endorsed by the publisher.
